# Deciphering the role of HPV-mediated metabolic regulation in shaping the tumor microenvironment and its implications for immunotherapy in HNSCC

**DOI:** 10.3389/fimmu.2023.1275270

**Published:** 2023-10-09

**Authors:** Xiangjin Gong, Jingwen Xiong, Yu Gong, Jieying Zhang, Jinhao Zhang, Guanhu Yang, Hao Chi, Gang Tian

**Affiliations:** ^1^ Department of Sports Rehabilitation, Southwest Medical University, Luzhou, China; ^2^ First Teaching Hospital of Tianjin University of Traditional Chinese Medicine, Tianjin, China; ^3^ National Clinical Research Center for Chinese Medicine Acupuncture and Moxibustion, Tianjin, China; ^4^ School of Stomatology, Southwest Medical University, Luzhou, China; ^5^ Department of Specialty Medicine, Ohio University, Athens, OH, United States; ^6^ Department of Clinical Medicine, School of Clinical Medicine, Affiliated Hospital of Southwest Medical University, Luzhou, China; ^7^ Department of Laboratory Medicine, The Affiliated Hospital of Southwest Medical University, Luzhou, China; ^8^ Department of Laboratory Medicine, Southwest Medical University, Luzhou, China; ^9^ Sichuan Province Engineering Technology Research Center of Molecular Diagnosis of Clinical Diseases, Luzhou, China; ^10^ Molecular Diagnosis of Clinical Diseases Key Laboratory of Luzhou, Luzhou, China

**Keywords:** HPV, HNSCC, tumor viruses, tumor viruses microenvironment, immunotherapy, markers

## Abstract

Head and neck squamous cell carcinoma (HNSCC), as a complex and variable malignancy, poses a significant threat to human health. Since the intricate association between HPV and HNSCC emerged, its role within the TME has garnered extensive attention. HPV+HNSCC exhibits distinct immunological characteristics within the TME, intricately intertwined with mechanisms of immune evasion. HPV employs multifaceted pathways to intervene in metabolic regulation within the TME, exerting influence over immune cell functionality and neoplastic cell genesis. Furthermore, the heightened immune reactivity exhibited by HPV+HNSCC within the TME augments responses to immune interventions such as immune checkpoint inhibitors. Therefore, amidst the current limitations of therapeutic approaches, immunotherapy stands as a promising strategy to overcome the conventional confines of treating HNSCC. This article comprehensively outlines the impact of HPV on the inception and progression of HNSCC while discussing the amalgamation of metabolic regulation within the TME and immunotherapeutic strategies. By intervening in the reciprocal interactions between HPV and HNSCC within the TME, the potential to modulate the efficacy of immune-based treatments becomes evident. Concurrently, a synthesis of pertinent biomarker development is summarized. Such endeavors hold paramount significance for personalized therapeutic approaches and the more effective management of HNSCC patients.

## 1 Background

Head and neck squamous cell carcinoma (HNSCC) ranks as the sixth most common malignant tumor globally, with a mortality rate of approximately 50% and a 5-year survival rate of only 40%-50% (1, 2). It predominantly affects the squamous epithelial cells in regions such as the mouth, nose, pharynx, and larynx. Clinical manifestations encompass masses, ulcers, hoarseness of voice, and may vary depending on the location of the occurrence. The etiology of HNSCC is associated with deleterious lifestyle habits, such as tobacco and alcohol abuse, as well as infection with human papillomavirus (HPV) (3).

The incidence of HPV+HNSCC is increasing yearly and has developed into a major pathogenic factor (4). HPV is a spherical non-enveloped double-stranded DNA virus, and its coding chain consists of an early transcription region, a late transcription region, and a non-coding region (5). The early transcription region encodes seven early proteins, among which E6 and E7 are the main oncogenic proteins. HPV exerts multiple effects within the tumor microenvironment (TME) of HNSCC. E5/7 suppresses the expression of major histocompatibility complex class I (MHC-I) on the cell surface, reducing the immune cells’ ability to recognize and attack tumor cells (6, 7). HPV-infected tumor cells release immune inhibitory factors such as transforming growth factor-β and interleukin-10 (IL-10), suppressing the activity and function of surrounding immune cells (8). Understanding the role of HPV in the TME of HNSCC can provide vital information for the development of more effective treatment strategies and preventive measures.

The conventional treatments for HNSCC mainly include surgical resection, radiotherapy, and chemotherapy (9). HNSCC often exhibits aggressive growth, making local control challenging with surgical resection. Radiotherapy and chemotherapy may cause damage to normal tissues in patients, leading to side effects. Hence, there exists an urgent imperative for novel and efficacious therapeutic modalities. Immunotherapy demonstrates remarkable specificity as it has the ability to activate the host’s immune system to target tumor cells. This approach circumvents the toxic side effects associated with conventional treatments and engenders a sustained anti-tumor immune response.

This article reviews how HPV in the TME affects the development of HNSCC and the mechanisms of HPV-immunotherapy interactions, weighs the integration of metabolic regulation of HPV in the TME with immunotherapeutic strategies, and summarizes the clinical applications of relevant biomarker development.

## 2 Regulatory mechanisms of HPV in HNSCC development

### 2.1 HPV accompanies the development of HNSCC

#### 2.1.1 E6 protein

One of the primary functions of E6 is to promote cell proliferation by inhibiting the cell cycle regulatory proteins p53 and pRb, disrupting the normal cell cycle regulation and leading to uncontrolled cell proliferation (10, 11). E6 binds to pRb, reducing its inhibition of the E2F transcription factor, thus propelling the cell cycle, promoting cell proliferation, and growth (12). Moreover, E6 is involved in the regulation of signaling pathways and acts in conjunction with the host cell oncogene ras in HNSCC, facilitating continuous transcription in cells (13). Another significant role of E6 is to inhibit apoptosis by interacting with key proteins in the host cells of HNSCC, disrupting the apoptotic signaling pathway. It accomplishes this by inhibiting the P300/CBP complex or through intrinsic apoptosis pathways, upregulating pro-apoptotic proteins (Bak and Bax) while inhibiting the anti-apoptotic protein Bcl2 (14). Additionally, E6 can employ the ubiquitin-dependent pathway to degrade p53, affecting cell apoptosis (15).

#### 2.1.2 E7 protein

The E7 protein specifically binds to the cullin2 ubiquitin ligase complex, releasing pRb’s inhibitory effect on the cell cycle (16). This activation leads to the liberation of E2F, promoting gene expression related to the S phase within the cell, thereby facilitating unrestricted cell proliferation and transformation. Additionally, it also induces cell cycle dysregulation by modulating cyclin-dependent kinases (CDK) or binding regulatory proteins like p21 and p27, thereby activating CDK2 (13). On the other hand, E7 interferes with several key apoptosis regulatory pathways within the cell. E7 can disrupt multiple intracellular signaling pathways, such as PI3K/Akt and Wnt/β-catenin, altering the balance between apoptotic and survival signals within the cell (17, 18). Some studies suggest that E7 expression may be linked to the sensitivity of HNSCC to chemotherapy drugs, leading to drug resistance in tumor cells (19). Interestingly, E7 induces the degradation of pRb, resulting in high levels of p16 expression. This inhibition of CDK4/6 and cyclin D interaction reduces cyclin D levels (20). As a consequence, this process drives host cells to undergo DNA repair, paradoxically increasing cancer cells’ responsiveness to radiotherapy and chemotherapy.

#### 2.1.3 E5 protein

E6/7 as pivotal carcinogenic factors in HPV infection, have been the focal point of research in HPV+HNSCC. Nevertheless, an increasing body of research suggests that E5 protein plays a significant role in the pathobiology of this disease. Its involvement in regulating host cell signaling, immune evasion, and cellular transformation mechanisms has garnered considerable attention. E5 impedes the signal transduction process of growth factor receptors in keratinocytes, inhibiting intracellular self-degradation and the growth and differentiation of keratinocyte cells. Consequently, this leads to malignant proliferation of epithelial cells, promoting early tumor development (21). In cervical cancer, E5 induces the more efficient entry of epidermal growth factor receptor (EGFR) into cell surfaces, accelerating the signaling of growth factors. Sung discovered that variations in E5 expression levels in HPV+HNSCC are correlated with EGFR expression, and both can serve as predictive markers for recurrence-free survival (22). However, further research is necessary to ascertain the specific mechanisms. Moreover, E5 employs E2F1 to induce elevated expression of CENPM, enhancing radiation sensitivity during HNSCC cell cycle redistribution, suggesting the potential clinical advantage of monitoring CENPM levels (23).

E5 can also inhibit apoptosis, contributing to the survival and dissemination of tumor cells. It interferes with the aggregation of Fas-related proteins with the death domain and suppresses Fas receptor expression, thereby disrupting the formation of death-inducing signal complexes and inhibiting TNF and FasL-related cell apoptosis-inducing ligands, decelerating the rate of cellular apoptosis (24). On another front, E5 employs a myriad of mechanisms to assist HPV+HNSCC in evading immune system surveillance and attacks. E5 downregulates the expression and transport of critical immune receptors such as MHC-I and MHC-II, influencing antigen presentation and modulating immune responses (25). Furthermore, E5 interferes with the interferon (IFN) signaling pathway, inhibiting IFN production and promoting immune evasion. Studies have reported that E5 achieves this by downregulating STAT1 expression, inhibiting the transcription of downstream interferon-stimulated genes (ISGs) (26). Alternatively, it directly inhibits the keratinocyte-specific expression of IFNκ, leading to ISG silencing. Interestingly, E5-mediated EGFR signaling pathways are implicated in influencing IFNκ expression. Furthermore, E5 induces cellular transformation and expedites carcinogenesis through the EGFR1 signaling pathway (PI3K-Akt signaling pathway and MAP kinase pathway) (27).

### 2.2 Role of HPV in HNSCC TME

**Figure 1:**
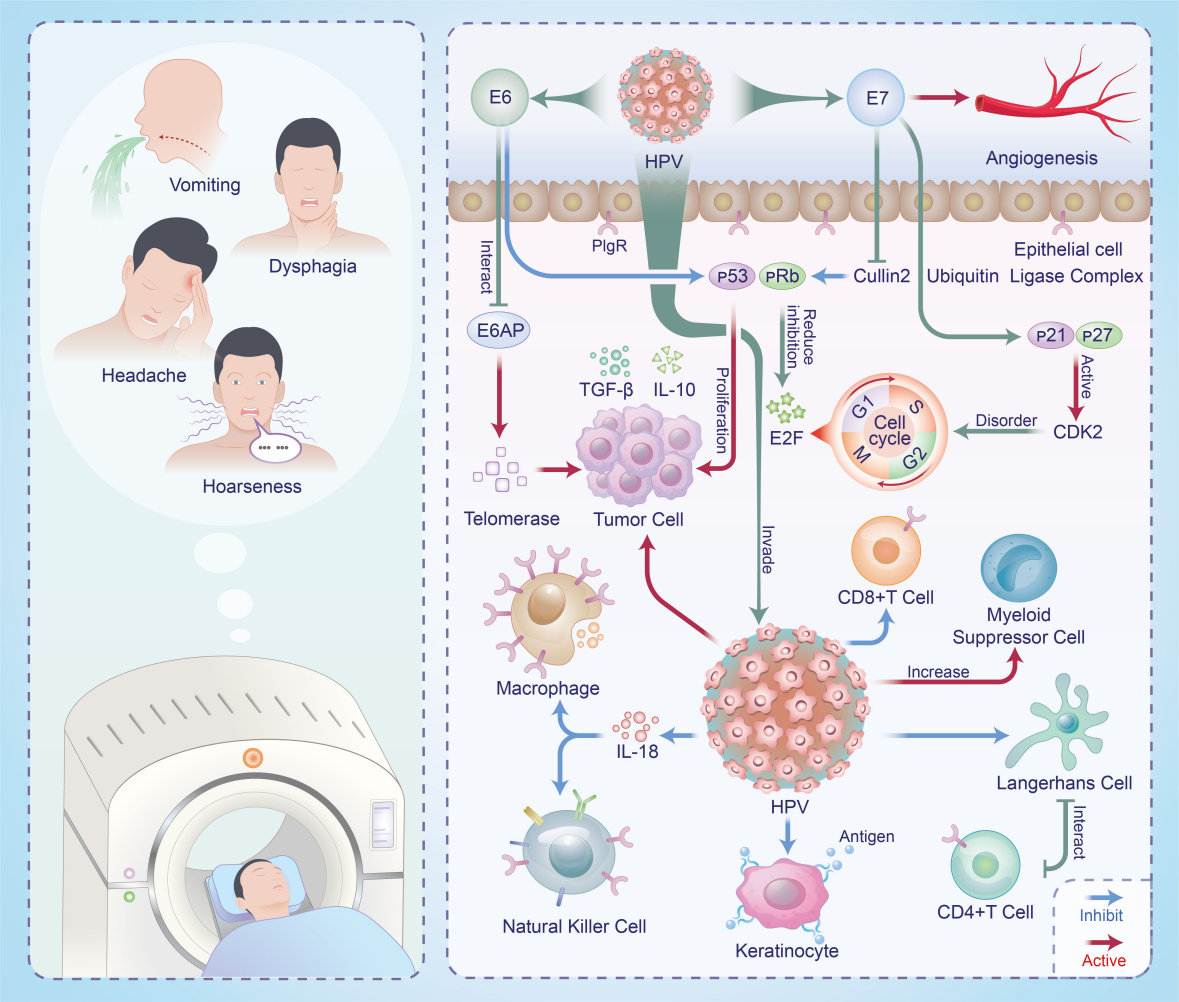
Mechanisms of interaction between HPV and HNSCC in TME and clinical manifestations in patients.

In the current field of HNSCC research, the role of TME is gaining increasing attention. Within the TME, there are intricate interactions between host cells and HPV, leading to various processes such as immune evasion, cell migration, epithelial-mesenchymal transition, and angiogenesis. These interactions, in turn, profoundly influence the growth, progression, and treatment response of HNSCC (**Figure 1**).

During the protracted process of HPV+HNSCC co-development, a series of multi-tiered and multi-level immune evasion mechanisms have emerged, encompassing both innate and adaptive immunity. Cell factors, chemokines, and complement constituents actively participate in constituting the organism’s natural immune system, including IFNs, tumor necrosis factor-alpha, IL-18, among others. HPV’s binding with p48 affects the formation and signal transduction of heterotrimeric complexes involving transcriptional activator STAT1, STAT2, and p48, leading to the disruption of IFN-stimulated response element binding and nuclear translocation, thereby inhibiting IFN activation (28). Research has revealed that HPV16 E6 competes with IL-18 for the IL-18 receptor alpha chain, suppressing IL-18 expression and IFN-y production, thereby inhibiting the phagocytic function of local tissue macrophages and their corresponding natural killer (NK) cell activity (29).

At the level of acquired immunity, HPV infection affects the antigen processing and presentation pathways, thereby reducing the efficiency of tumor antigen presentation to immune cells. HPV acts upon the MHC-I heavy chain, downregulating certain gene promoters (such as antigen processing-related transporter 1 and proteasome subunit low molecular weight peptide 2), leading to transcriptional activation inhibition. This, in turn, hinders effective binding of viral antigens to MHC-I peptide-binding grooves, redirecting them towards the Golgi apparatus and endoplasmic reticulum for presentation in the keratinocytes, subsequently decreasing the capture rate of viral antigens (30, 31). On the other hand, E7 interacts with antigenic peptide transfer proteins, inhibiting the presentation of antigens by keratinocytes and disrupting cellular immune responses (30). Langerhans cells (LC) also play a crucial role in bridging innate and acquired immunity through their antigen-presenting function (32). LCs located in the basal layer of the epidermis have limited access to low-level expression of HPV early genes, mainly found in the upper layers of the epidermis, resulting in a significantly reduced antigen presentation rate. Furthermore, the interaction between LCs and specific CD4+ T cells is also hampered by HPV, resulting in enhanced adaptive cytotoxic T lymphocyte responses. The increased presence of virus-induced immunosuppressive cells (such as regulatory T cells and myeloid-derived suppressor cells) may inhibit the activity of CD8+ T cells, thus avoiding immune system attacks (33).

Another immune escape pathway involves the binding of tumor cells’ surface-programmed death ligand-1 (PD-L1) with immune cells’ programmed cell death protein-1 (PD-1) (34, 35). Data analysis has revealed an interesting phenomenon, where highly active B lymphocytes in HPV+HNSCC patients significantly influence the prognosis (36). Moreover, substantial differences are observed in the immune cell composition of TME between positive and negative patients, notably in terms of CD4+ T cell aggregation and infiltration. Hence, analyzing the differences in patients’ immune cell composition can aid in selecting more effective immunotherapy strategies and predicting patient outcomes more accurately.

HPV induces tumor cells to undergo epithelial-mesenchymal transition, activating transcription factors like ZEB1/2 and Slug, leading them to transform from adherent epithelial cells into migratory tumor cells with mesenchymal characteristics, thereby enhancing their migratory and invasive capabilities (37). Subsequently, HPV stimulates the release of inflammatory factors within the TME, further promoting cancer cell dissemination. The increased formation of neovascularization in the TME facilitates tumor spread (38). HPV stimulates neointimal formation by modulating cell signaling pathways, enhancing vascular endothelial growth factor expression or directly inhibiting angiogenesis inhibitory factor, which in turn stimulates neointimal formation. Additionally, HPV can release chemical and cellular factors, creating a TME that is more conducive to new blood vessel growth and expansion. Meta-analysis has revealed that even under high oxygen conditions, HPV-infected tumor cells still preferentially utilize glucose metabolism, a phenomenon known as the Warburg effect (39). This metabolic adaptation allows tumor cells to gain a growth advantage in glycolysis and provide more carbon sources for HNSCC growth.

Indeed, it is important to note that HPV’s effects may vary among different individuals and tumor types, thus the relative significance of these mechanisms can vary depending on the specific context.

## 3 HNSCC immunotherapy

### 3.1 Mechanisms of HPV-immunotherapy interactions

In certain cases, HPV induces tumor cells to express more immunogenic antigens, thereby enhancing the immune system’s ability to attack cancer cells, which is considered a potential advantage for immunotherapy. This advantage may be attributed to the unique TME formed by HPV, including E6/7, CD4+/8+ T cells, which bolster immune cell surveillance of tumor cells. HPV16 E6 has been found to elicit specific T cell responses and CD8+ T cell responses in HNSCC patients, optimizing the clinical response to standard treatments (40). Consequently, recruiting HPV16-specific T cells can lead to better prognoses and improved patient treatment outcomes. The quantity of tumor-infiltrating lymphocytes (TILs) is commonly used to assess adaptive immune responses. Comprehensive analysis of numerous cases has revealed that HPV+HNSCC patients with high survival rates often exhibit high expression of TILs, particularly CD8+ T cells (41). Co-stimulatory T cell receptor agonists (such as CD137) are emerging as a novel approach in HNSCC immunotherapy. They accelerate antigen-presenting cell capture and processing of the abundant antigens released upon HPV infection. Studies have shown that positive patients treated with cetuximab in combination with urelumab exhibit elevated CD137 levels in NK cells, which is beneficial for immunotherapy (42). Indeed, novel fusion proteins like CUE-101 hold great promise as potential therapeutic approaches in HNSCC immunotherapy. CUE-101 can promote antigen presentation, activate immune responses, and enhance the immune system’s memory effect on tumor cells. Phase I clinical trials have found that CUE-101 activates HPV16 E7 tumor-specific T cells, subsequently generating dose-dependent cytokines that inhibit HNSCC growth (43). E7 presents a broad prospect for specific immunotherapy, with CUE-101 being used as a second-line monotherapy for HPV+HNSCC or in combination with pembrolizumab.

However, HPV infection can also negatively impact immunotherapy, such as immune evasion or activation of immune inhibitory pathways, thereby weakening the effectiveness of immune-based treatments. A fundamental and effective immunotherapy strategy for HPV+HNSCC involves the use of anti-PD-1/PD-L1 antibodies (44). The study identified HPV-specific regulation of signaling pathways, pointing out that miRNA-mRNA interactions in positive HNSCC are interconnected with the PD-1 checkpoint pathway, PD-L1 expression (45). Antibody immunotherapy inhibits tumor growth and spread by blocking the binding of PD-1 to PD-L1, lifting T-cell suppression and restoring immune activity. Meta-analyses have shown that positive patients treated with antibody immunotherapy experience lower mortality rates compared to negative patients, and PD-L1 can serve as a predictive marker for immunotherapy response (46, 47). However, a drawback is that these drugs have low efficacy in a significant proportion of patients who have failed chemotherapy, and the low TIL characteristics observed in negative patients contribute to increased treatment failure rates (48). Researchers propose that activating the immune system of recurrent HNSCC patients through adoptive transfer of antigen-specific cells or vaccination may be more effective (49). T cell-enhancing vaccines can activate host T cells and stimulate the formation of long-term immune memory, hastening the recognition and attack of HPV-infected cells and inhibiting immune evasion. Currently, exploring the use of cytotoxic T-lymphocyte-associated antigen 4 (CTLA-4) inhibitors to enhance the immune response to HPV infection is underway (50). This inhibitor inhibits HPV-induced generation of aberrantly expressed CTLA-4 binding to CD80/86 and enhances T cell activation and attack. Additionally, personalized immunotherapy offers patients more precise and effective treatment outcomes. Chimeric antigen receptor T cell therapy (CAR-T) activates T cell attack mechanisms by binding the antigen recognition structure on CAR to specific antigens present on the surface of HPV-infected cells (51, 52). CAR-T cell therapy for HPV+HNSCC patients requires careful monitoring of immune responses and management of adverse reactions due to the complexity of the patients’ immune status.

### 3.2 Development of relevant markers

Early diagnosis of HPV+HNSCC patients can prevent tumor dissemination to lymph nodes and distant organs, thus reducing psychological burdens and enhancing treatment success rates. Common diagnostic modalities include p16 protein examination or HPV DNA testing in tissue samples (53). Certain serum biomarkers, such as TP53 antibodies and CYFRA 21-1, hold value in early adjunctive diagnostics (54–56). The tumor suppressor gene TP53 encodes the p53 protein, crucial for genomic stability maintenance. In HNSCC patients, p53 mutations alter protein structure, prompting the immune system to produce antibodies against aberrant p53 protein. Detection of serum antibody levels aids in the early detection of abnormal p53 protein expression. Elevated levels of the cytokeratin complex CYFRA 21-1 in serum result from HNSCC cell proliferation, thus rendering it one of the markers for early diagnosis and monitoring. Moreover, EGFR and certain specific gene or transcriptome characteristics are associated with early diagnosis of HPV+HNSCC. Immunotherapeutic biomarkers have the capacity to mirror the immunological status of patients, anticipate treatment responses, and facilitate the refinement of therapeutic approaches (57). Presently, biomarker development predominantly revolves around T-cell functionality, genomics, and transcriptomics analysis, immune checkpoint expression, and inflammatory factors (58–61). For instance, the T-cell-related inflammatory gene expression profile and tumor mutation burden can predict the response of HNSCC to pembrolizumab (**Table 1**) (81).

**Table 1:** HNSCC markers.

Name	Source	Role	Purpose	Literatures
TP53	Serum	TP53 mutations lead to loss of tumor suppressor p53 function	Early diagnostic and prognostic markers	(62)
MPS-1	Serum	High sensitivity and specificity	Early diagnostic markers	(63)
VEGF-C	Serum	Induces endothelial cell proliferation, migration and survival	Transfer marker	(64)
CYFRA 21-1	Serum	High levels of CYFRA 21-1 suggest the presence of HNSCC	Early diagnostic markers	(65)
Midkine	Serum	Midkine promotes tumor-specific functions	Prognostic marker	(66)
COX-2	Cell	COX-2 upregulates fibronectin to promote HNSCC metastasis	Predictive marker	(67)
EGFR	Cell	EGFR mediates HNSCC metastasis	Prognostic marker	(68)
Cyclin D1	Cell	Cyclin D1 amplification is associated with HNSCC invasion	Prognostic marker	(69)
CDK19	Cell	Influences gene transcription as a signaling pathway coactivator	Predictive markers for relapse	(70)
NAT10	Cell	NAT10 regulates tumor cell proliferation and migration	Prognostic marker	(71)
TRIM21	Cell	Associated with immune cell infiltration of the primary tumor	Prognostic marker	(72)
SHOX2	DNA	SHOX2 hypermethylation promotes HNSCC invasion and metastasis	Prognostic marker	(73)
SEPT9	DNA	SEPT9 methylation is associated with HNSCC lymph node metastasis	Prognostic marker	
has-mir-383	miRNA	Extensive involvement in tumorigenesis	Diagnostic and prognostic markers	(74)
has-mir-615	miRNA	Specific expression indicates HNSCC development or prognosis	Diagnostic and prognostic markers	
has-mir-877	miRNA	Specific expression indicates HNSCC development or prognosis	Diagnostic and prognostic markers	
miR-125a	miRNA	Affects cell growth and differentiation	Diagnostic marker	(75)
miR-200a	miRNA	Influence on stress and immune response	Diagnostic marker	
circPVT1	circRNA	Influencing the malignant phenotype of tumor cell lines	Prognostic marker	(76)
circCORO1C	circRNA	Influence on tumor progression	Prognostic marker	
circ_0000199	circRNA	High expression increases tumor recurrence and mortality	Prognostic marker	
circCUX1	circRNA	Influence on tumor size and distant metastasis	Prognostic marker	
circPARD3	circRNA	Associated with HNSCC staging	Prognostic marker	
YRNA	sncRNA	Associated with advanced cancer staging	Biomarker	(77)
NR2F6		Regulation of cytokine expression, immunotherapy targets	Prognostic marker	(78)
CXCR4		Increases metastasis and decreases overall survival	Transfer marker	(79)
CCR7		Control of immune cell responses to inflammatory stimuli	Predictive markers of metastasis	(80)

MPS-1, Metallopanstimulin-1; VEGF, Vascular endothelial growth factor ; COX-2, Cyclooxygenase-2; EGFR, Epidermal growth factor receptor; NR2F6, Nuclear receptor subfamily 2, group F, member 6; CDK, Cyclin dependent kinase; NAT10, N-acetyltransferase 10; TRIM21, Tripartite motif containing 21; CXCR4, CXC chemokine receptor 4; CCR7,CC chemokine receptor 7.

## 4 Discussion

Studying the interaction mechanisms between HPV and the TME in HPV+HNSCC patients holds significant importance for cancer pathogenesis and treatment. However, current research in this area still has some limitations. While the incidence of HPV+HNSCC is gradually increasing in certain regions, such as the United States, its relative rarity poses a challenge in acquiring a sufficient number of clinical specimens for in-depth research and statistical analysis. The complexity of TME also limits our understanding of the interactions and regulatory mechanisms of its various components. Immunotherapy has bridged the gaps left by conventional treatments, showing promise in promoting personalized therapies and reducing treatment toxicity. Despite exploring personalized treatments and associated biomarkers, a clinically effective approach remains absent. In-depth research on HPV+HNSCC TME and the identification of biomarkers within the TME may pave the way for achieving personalized and effective immunotherapy.

## Author contributions

XG: Conceptualization, Data curation, Writing – original draft. JX: Writing – original draft. YG: Writing – original draft. JieZ: Writing – original draft. JinZ: Writing – original draft. GY: Conceptualization, Writing – review & editing. HC: Conceptualization, Writing – review & editing. GT: Conceptualization, Writing – review & editing.
